# Khz (Fusion of *Ganoderma lucidum* and *Polyporus umbellatus* Mycelia) Induces Apoptosis by Increasing Intracellular Calcium Levels and Activating JNK and NADPH Oxidase-Dependent Generation of Reactive Oxygen Species

**DOI:** 10.1371/journal.pone.0046208

**Published:** 2012-10-08

**Authors:** Tae Hwan Kim, Ju sung Kim, Zoo haye Kim, Ren Bin Huang, Ren Sheng Wang

**Affiliations:** 1 Department of Radiotherapy, The First Affiliated Hospital, Guangxi Medical University, Nanning, China; 2 Clinical Medicine, Harbin Medical University, Harbin, China; 3 Graduate School of Information Science, Nagoya University, Nagoya, Japan; 4 Graduate School of Pharmacology, Guangxi Medical University, Nanning, China; Indiana University School of Medicine, United States of America

## Abstract

Khz is a compound derived from the fusion of *Ganoderma lucidum* and *Polyporus umbellatus* mycelia that inhibits the growth of cancer cells. The results of the present study show that Khz induced apoptosis preferentially in transformed cells and had only minimal effects on non-transformed cells. Furthermore, Khz induced apoptosis by increasing the intracellular Ca^2+^ concentration ([Ca^2+^]*_i_*) and activating JNK to generate reactive oxygen species (ROS) *via* NADPH oxidase and the mitochondria. Khz-induced apoptosis was caspase-dependent and occurred *via* a mitochondrial pathway. ROS generation by NADPH oxidase was critical for Khz-induced apoptosis, and although mitochondrial ROS production was also required, it appeared to occur secondary to ROS generation by NADPH oxidase. Activation of NADPH oxidase was demonstrated by the translocation of regulatory subunits p47^phox^ and p67^phox^ to the cell membrane and was necessary for ROS generation by Khz. Khz triggered a rapid and sustained increase in [Ca^2+^]*_i_*, which activated JNK. JNK plays a key role in the activation of NADPH oxidase because inhibition of its expression or activity abrogated membrane translocation of the p47^phox^ and p67^phox^ subunits and ROS generation. In summary, these data indicate that Khz preferentially induces apoptosis in cancer cells, and the signaling mechanisms involve an increase in [Ca^2+^]*_i_*, JNK activation, and ROS generation *via* NADPH oxidase and mitochondria.

## Introduction

Cancer develops from abnormal cellular proliferation or defects in apoptosis that lead to uncontrolled growth [1]. Therefore, new treatments that target the proliferation and apoptosis of cancer cells are necessary. Under normal conditions, programmed cell death occurs after exposure to pathological factors. Apoptosis involves cell shrinkage, condensation of nuclei and chromatin, and DNA fragmentation, all of which leave a cell with unmistakable morphological characteristics. Apoptosis is initiated by external signals through a series of cysteine acid proteases, including important regulatory factors such as caspases. Defective signaling in the regulation of cell death can result in the abnormal proliferation of cells and can cause cancer. Thus, repairing the defective cell death mechanisms or using drugs or food components that induce cell differentiation may be a promising approach for the development of anticancer agents [2], [3]. In particular, many studies are being performed to identify natural products that can be used as anticancer drugs and that do not have the toxicity and side effects associated with chemotherapeutic drugs. Indeed, several biologically active ingredients that show effective anticancer activity have been derived from edible or medicinal mushrooms [4]–[6]. The anticancer effects of *Ganoderma lucidum* have been described in various studies [7]–[10]. *Polyporus umbellatus* induces G2/M cell cycle arrest and the induction of apoptosis in HepG2 cells, thereby causing growth suppression [11].

**Figure 1:**
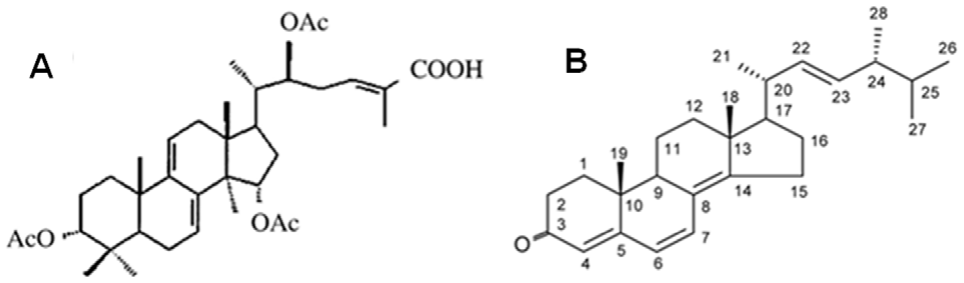
(A) Structure of ganoderic acid T (GA-T). (B) Chemical structure of the compound isolated from the sclerotia of *Polyporus umbellatus*.

**Figure 2:**
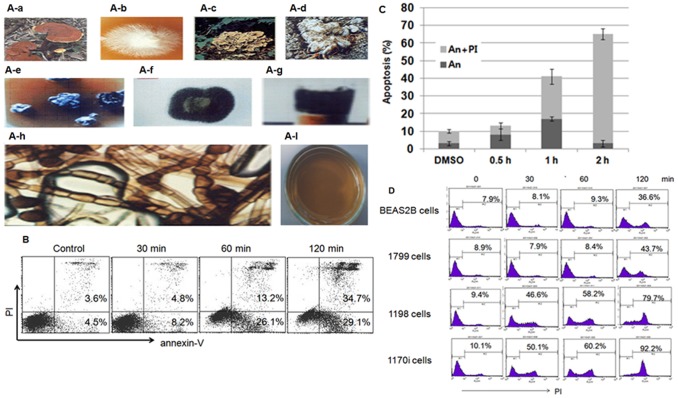
Khz induces apoptosis in transformed cells. (A) (A-a) The shape and type of fused fruiting bodies. (A-b) Hyphae isolated from a *Ganoderma lucidum* mushroom on a Petri dish. (A-c) Shape of *G. lucidum*. (A-d) Shape and type of fused fruiting bodies and hyphae from *Polyporus umbellatus*. (A-e) Fusion of *G. lucidum* and *P. umbellatus*. (A-f) The fused hyphae of *G. lucidum* and *P. umbellatus*. (A-g) Agar-cultured fusion fungi. (A-h) DNA from fused hyphae (Khz). (A-i) Cultivation conditions for Khz. (B) Analysis of apoptosis using annexin-V-FITC (An) and propidium iodide (PI) staining. HepG2 cells were treated with a 1∶2 dilution of Khz, and apoptosis was analyzed after 0.5, 1, and 2 h by flow cytometry. Data are representative of more than 3 experiments. (C) HepG2 cells were treated with a 1∶2 dilution of Khz and stained with An and PI for flow cytometric analysis. Data represent mean ± SD. (D) BEAS-2B, 1799, 1198, and 1170-I cells were treated with Khz (1∶2 dilution), and apoptosis was examined by PI staining followed by flow cytometric analysis. Data are representative of more than 3 experiments.

Khz is an extract mixture from the mycelia of a *G. lucidum* (Figure 1A) and *P. umbellatus* (Figure 1B) nuclear fusion (Figure 2A). The anticancer effect of the fusion of *G. lucidum* and *P. umbellatus* has been previously demonstrated. In this study, we investigated the mechanism underlying Khz-induced cell death in hepatoma cells.

Oxidative stress is widely implicated in apoptotic and non-apoptotic cell death [12]–[14]. Major sources of intracellular reactive oxygen species (ROS) include NADPH oxidase and the mitochondrial electron transport chain (ETC). When ROS production, either by the mitochondria or by NADPH oxidase, becomes excessive, the natural cellular antioxidant defense system is overwhelmed, which results in oxidative stress. Cancer cells are more susceptible to oxidative stress than “normal” cells, and some anticancer agents such as cisplatin, arsenic trioxide (As_2_O_3_), and 2-methoxyestradiol exert their effects by inducing ROS production [15]–[17]. Much of the available data indicate that the ROS that accumulate during the process of cell death are generated by the mitochondria in response to impairment of the mitochondrial respiratory chain [18]–[21]. Although mitochondrial ROS production is regarded as an integral component of the apoptotic program, the role of NADPH oxidase as a primary source of ROS during the induction of apoptosis has also been reported [22]–[24]. Sustained elevation of the intracellular Ca^2+^ concentration ([Ca^2+^]*_i_*) is associated with the induction of apoptosis [25]. When the cytoplasmic [Ca^2+^]*_i_* increases, the mitochondria take up Ca^2+^ and function as a Ca^2+^-buffer; however, excessive accumulation of mitochondrial Ca^2+^ triggers apoptosis, at least in part, by inducing ROS generation *via* the mitochondrial ETC. An increase in cytoplasmic [Ca^2+^]*_i_* can also activate NADPH oxidase, which has been well documented in neutrophils [26]. In some cell types, the activation of protein kinase C *via* intracellular Ca^2+^ leads to the phosphorylation of the p47^phox^ subunit and subsequent enzyme assembly [27].

In the present study, we investigated the role of Khz in cellular apoptosis and found that Khz induced a sustained increase in [Ca^2+^]*_i_* that resulted in ROS generation by NADPH oxidase *via* JNK and, finally, cellular apoptosis.

## Materials and Methods

### Cell lines and Khz treatment

The BEAS-2B (normal immortalized), 1799 (non-transformed), 1198 (transformed but non-tumorigenic), and 1170-I (tumorigenic) cell lines that compose an *in vivo* lung carcinogenesis model have been previously described [28], [29]. The human liver cancer cell line HepG2 was maintained in RPMI 1640 media supplemented with 10% fetal bovine serum, 100 U/ml penicillin G sodium, 100 µg/ml streptomycin sulfate, and 0.25 µg/ml amphotericin B. Unless otherwise indicated, all cells were treated with Khz diluted 1∶2 in the media.

### Extraction of Khz (Fusion of *G. lucidum* and *P. umbellatus* mycelia)

First, 1 kg of powder was added to 8.5 L of clean water, heated to 115°C, and extracted for 60 min under pressure. This was followed by a 60-min maturation period and hydraulic crossroad gathering. Next, the remaining water from the first extraction was added to 7.5 L of clean water, heated to 115°C, extracted under pressure for 60 min, matured for a further 60 min, and subjected to hydraulic crossroad gathering. The first and second extracts were then mixed, boiled, and placed in bottles after 5 min.

### Reagents and antibodies

Mitochondria-targeted ubiquinone (MitoQ) is an ubiquinol antioxidant attached to a lipophilic triphenylphosphonium (TPP) cation [30]. MitoQ and TPP were kind gifts from Dr. Michael P. Murphy (Medical Research Council Dunn Human Nutrition Unit, UK). SP600125, apocynin, and cyclosporin A (CsA) were purchased from Calbiochem (San Diego, CA, USA), and N-acetyl cysteine (NAC) and ethylene glycol tetraacetic acid (EGTA) were purchased from Sigma (St. Louis, MO, USA). z-VAD-fmk was obtained from R&D Systems (Minneapolis, MN, USA), diphenylene iodonium (DPI) was from Cayman Chemical (Ann Arbor, MI, USA), and BAPTA-AM was from Invitrogen (Eugene, OR, USA).

Antibodies against JNK (sc-571), p47^phox^ (sc-14015), p67^phox^ (sc-15342), caspase 3 (sc-7148), PARP (sc-7150), and cytochrome c (sc-13561) were purchased from Santa Cruz Biotechnology (Santa Cruz, CA, USA). Anti-p-JNK (9255) antibodies were obtained from Cell Signaling (Danvers, MA, USA), and a COX IV antibody (A21347) was obtained from Invitrogen.

### Western blot analysis

Cells were lysed in an extraction buffer (31.25 mM Tris-HCl [pH 6.8], 1% sodium dodecyl sulfate [SDS], 10% glycerol, and 2.5%-mercaptoethanol), and the whole cell lysates were subjected to SDS-polyacrylamide gel electrophoresis. Size-fractionated proteins on the gel were transferred onto a nitrocellulose membrane. The membrane was blocked in 5% skim milk in Tris-buffered saline containing 0.05% Tween 20 and incubated with a primary antibody. After washing, the membrane was incubated with the peroxidase-conjugated secondary antibody. The protein band of interest was detected using enhanced chemiluminescence reagents (Amersham).

### Apoptosis assay

Cells treated with Khz were washed twice in cold phosphate-buffered saline (PBS) and stained with annexin-V-FITC (A13199; Invitrogen) and propidium iodide (PI) according to the manufacturer's instructions. Briefly, annexin-V-FITC (5 µL) was added to the cells, which were resuspended in 100 µL of binding buffer (10 mM HEPES, 140 mM NaCl, and 2 mM CaCl_2_; pH 7.4). The cells were then incubated at room temperature for 15 min, and PI was added before flow cytometry or fluorescence microscopy analysis.

### Assessment of cytoplasmic and mitochondrial ROS levels

The levels of cytoplasmic ROS were estimated using the oxidation-sensitive fluorescent dye H_2_DCF-DA (2′,7′-dichlorodihydrofluorescein diacetate; Invitrogen) or the Amplex Red hydrogen peroxide assay kit (Invitrogen). For DCF staining, cells were loaded with H_2_DCF-DA (100 nM) for 1 h at 37°C and washed once with PBS. After treatment with Khz, ROS levels were analyzed using a flow cytometer (FACSCalibur, Becton Dickinson, San Jose, CA, USA) or a fluorescence microscope (Eclipse 80i; Nikon, Tokyo, Japan). The Amplex Red hydrogen peroxide assay was performed according to the manufacturer's protocol. In brief, cells were lysed in 50 µM Amplex Red solution supplemented with 0.1 U/mL horseradish peroxidase and incubated in the dark for 30 min. Fluorescence was measured in a plate reader (Victor 2, Perkin-Elmer Life Sciences, Boston, MA, USA) with an excitation wavelength of 540 nm and an emission wavelength of 590 nm.

Mitochondrial superoxide anion levels were analyzed by staining with MitoSOX™ Red (Invitrogen). Cells were loaded with MitoSOX Red (5 µM) for 30 min at 37°C and then treated with Khz. Fluorescence was then analyzed by flow cytometry or fluorescence microscopy.

### Preparation of subcellular fractions

To prepare the mitochondrial and cytosolic fractions, the cells (1×10^7^) were washed once in PBS and disrupted by passing them through a glass homogenizer 80 times in ice-cold isolation buffer (250 mM sucrose, 20 mM HEPES, 10 mM KCl, 1.5 mM MgCl_2_, 1 mM EGTA, 1 mM EDTA, 1 mM DTT, and 0.1 mM PMSF). Nuclei and undisrupted cells were removed by centrifugation at 750×*g* for 20 min at 4°C. The supernatant was further centrifuged at 10,000×*g* for 15 min at 4°C to obtain a mitochondria-enriched pellet and a cytoplasm-enriched supernatant.

Membrane and cytosolic fractions were prepared using the Compartmental Protein Extraction kit (Millipore, Temecula, CA, USA) according to the manufacturer's instructions.

### Ca^2+^ imaging

Digital imaging microscopic analysis of intracellular free Ca^2+^ was performed using the dye, fura-2 AM (Invitrogen). When fura-2 binds to Ca^2+^, its maximal absorption wavelength shifts from 363 to 335 nm. HepG2 cells (1×10^4^) were cultured in 35-mm glass-bottomed dishes and loaded with fura-2 AM (2 µM) for 30 min at 37°C. Fluorescence images of fura-2 were digitally captured at excitation wavelengths of 340 and 380 nm and at an emission wavelength of 510 nm using an IX70 fluorescence microscope (Olympus, Tokyo, Japan) equipped with a digital cooled charge-coupled device camera. Paired 340/380 ratiometric images were analyzed using Metafluor software (Molecular Devices, Sunnyvale, CA, USA). Confocal images of intracellular free Ca^2+^ were obtained using the Ca^2+^-sensitive fluorescent dye, fluo-4 AM (Invitrogen). Cells were loaded with fluo-4 AM (1 µM) for 30 min at 37°C, and Ca^2+^ imaging was performed using a confocal laser scanning microscope (LSM510; Carl Zeiss, Jena, Germany).

### Transfection of siRNA and plasmids

HepG2 cells were transfected with siRNA using Lipofectamine 2000 (Invitrogen) as described previously. The coding strand sequences of the siRNAs were as follows: 5′-CUG GUA UGA UCC UUC UGA AdTdT-3′ (JNK1), 5′-GAG GUA UAC ACA UAC UGA dTdT-3′ (Nox2), 5′-CUG UUG UGG ACC CAA UUC AdTdT-3′ (Nox4), and 5′-GUU CAG CGU GUC CGG CGA GdTdT-3′ (GFP). Bcl-2 cDNA was transfected into cells using the Lipofectamine-PLUS reagent (Invitrogen) according to the manufacturer's instructions. Stably transfected cells were selected using G418 (3 mg/mL).

## Results

### Khz induces apoptosis in transformed cells

Khz are shown in Fig. 2a. The aim of the present study was to examine whether Khz causes apoptosis in human cancer cells and, if so, to identify the signaling mechanisms involved. As shown in Fig. 2b and c, staining with annexin-V-FITC and PI revealed that Khz triggered apoptosis in HepG2 cells (annexin-V single-positive or annexin-V/PI double-positive cells) as early as 60 min after treatment. As apoptosis progressed, the population of cells stained with annexin-V alone declined, whereas that of the cells stained with both annexin-V and PI increased (Fig. 2c). The population of cells positive for PI alone may represent necrotic cells. The induction of preferential apoptosis by Khz in transformed cells was then evaluated in a series of cell lines that comprise an *in vivo* lung epithelial carcinogenesis model. BEAS-2B is an immortalized normal human bronchial epithelial cell line, and 1198 and 1170-I are transformed cell lines derived from BEAS-2B cells exposed *in vivo* to beeswax pellets containing cigarette smoke condensate (CSC). The 1799 cell line is a non-transformed line derived from BEAS-2B cells exposed to beeswax alone. Khz induced apoptosis in the transformed 1198 and 1170-I cells but not in non-transformed BEAS-2B and 1799 cells (Fig. 2d). These data indicate that Khz induces apoptosis preferentially in cancer cells, which suggests that it has potential as a cancer therapeutic agent.

### Khz-induced apoptosis is caspase-dependent and occurs through a mitochondrial pathway

**Figure 3:**
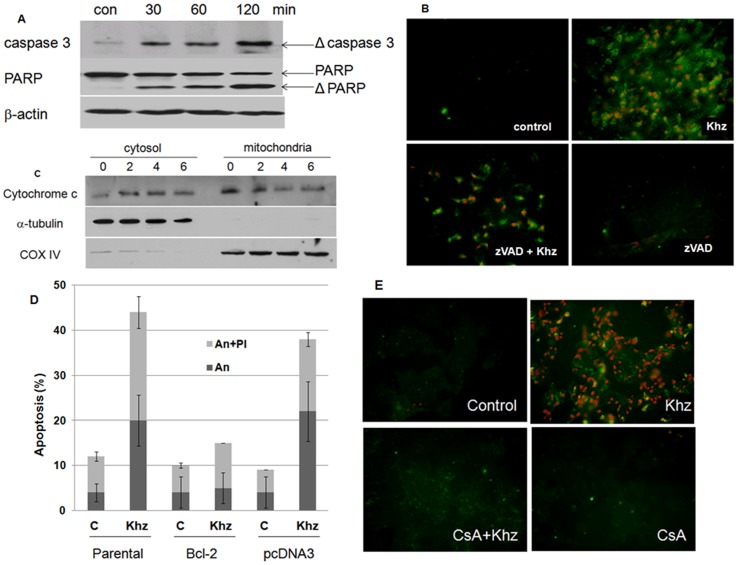
Khz induces apoptosis through a mitochondrial pathway. (A) HepG2 cells were treated with a 1∶2 dilution of Khz, and caspase or PARP activation was analyzed by immunoblotting. (B) Cells were pretreated with z-VAD-fmk (20 µM) for 1 h and treated with Khz at the same concentrations as seen in Fig. 2(B). After 1 h, cells were stained with propidium iodide (PI) and annexin-V-FITC (An) for fluorescent microscopy. (C) Immunoblot analysis of cytochrome c levels in cytosolic and mitochondrial fractions of HepG2 cells treated with Khz. (D) HepG2 cells stably transfected with Bcl-2 cDNA or an empty vector (pcDNA3) were treated with Khz for 1 h. Apoptosis was analyzed as in Fig. 2(C). (E) Cells were pretreated with CsA (10 µM) for 1 h. Khz treatment and analysis of apoptosis were performed as in Fig. 2(B). After 1 h, the cells were stained with PI and An for fluorescent microscopy.

To determine whether the Khz-induced apoptosis was caspase-dependent, caspase activation was analyzed after Khz treatment. Cleavage of caspase 3 and PARP (indicating their activation) increased in HepG2 cells after Khz treatment (Fig. 3a). Moreover, pretreatment of these cells with the pan-caspase inhibitor z-VAD-fmk completely blocked Khz-induced apoptosis (Fig. 3b), which indicates that Khz induces caspase-dependent apoptosis.

Natural products or cytotoxic chemicals often induce apoptosis through a mitochondrial pathway; therefore, the release of cytochrome c from the mitochondria into the cytosol was analyzed to examine whether Khz-induced apoptosis also occurred *via* a mitochondrial pathway. As shown in Fig. 3c, cytochrome c levels in the cytosol of HepG2 cells increased after Khz treatment, whereas cytochrome c levels in the mitochondria concurrently decreased, which indicates the release of mitochondrial cytochrome c. Ectopic expression of the protective Bcl-2 protein prevented Khz-induced apoptosis in HepG2 cells (Fig. 3d). Furthermore, CsA treatment (which blocks PTP opening by binding to cyclophilin D) abrogated Khz-induced apoptosis, which suggests that mitochondrial permeability transition was required for apoptosis (Fig. 3e).

### Oxidative stress mediates Khz-induced apoptosis

**Figure 4:**
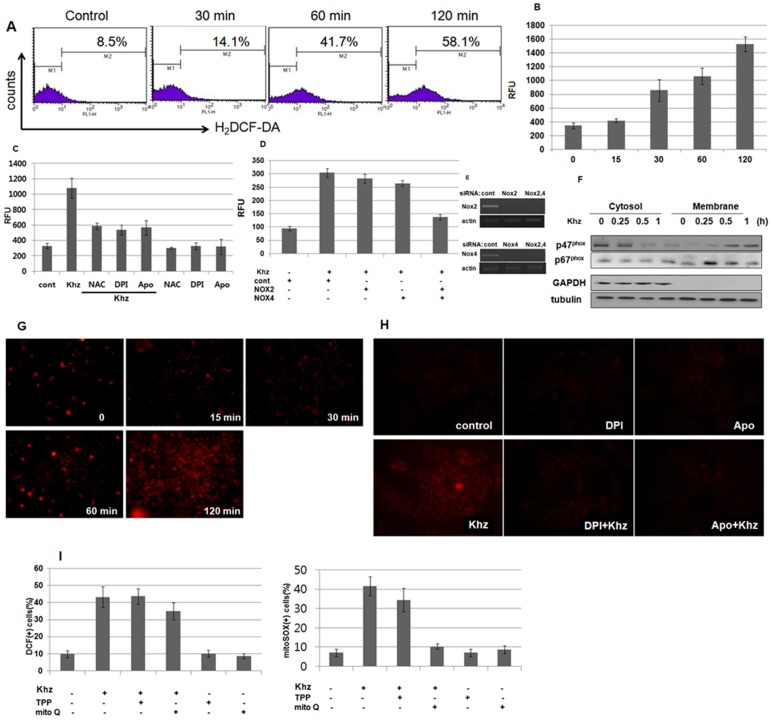
Khz triggers cytoplasmic and mitochondrial ROS generation. (A) HepG2 cells were loaded with H_2_DCF-DA and treated with Khz (diluted 1∶2), and the cytoplasmic ROS levels were assessed by flow cytometry. Data are representative of more than 3 experiments. (B) Intracellular ROS levels in HepG2 cells treated with Khz (diluted 1∶2) were analyzed using an Amplex Red hydrogen peroxide assay. Data represent mean ± SD. (C) Cells were pretreated with NAC (5 mM), DPI (10 µM), or apocynin (Apo; 300 µM) for 1 h. Intracellular ROS levels in HepG2 cells were analyzed 30 min after Khz treatment using the Amplex Red hydrogen peroxide assay. (D–E) HepG2 cells were transfected with siRNAs targeting Nox2 and Nox4. After 48 h, the cells were treated with Khz, and ROS generation was measured after 60 min using the Amplex Red hydrogen peroxide assay. Data represent mean ± SD. (E). Silencing of Nox2 and Nox4 mRNA expression by siRNA transfection was assessed by RT-PCR. (F) HepG2 cells were treated with Khz, and the membrane and cytosol fractions were separated using the Compartmental Protein Extraction kit (Millipore). The expression of the p47^phox^ and p67^phox^ proteins in the membrane and cytosol fractions was analyzed by immunoblotting. (G) HepG2 cells were loaded with MitoSOX Red for 30 min and treated with Khz. Mitochondrial ROS generation was then assessed by fluorescent microscopy at the indicated time points. Data are representative of more than 3 experiments. (H) HepG2 cells were pretreated with DPI or apocynin for 1 h and mitochondrial ROS generation was analyzed 60 min after Khz treatment as in (G). (I) HepG2 cells were pretreated with 0.5 µM MitoQ or TPP for 30 min. Right panel: Cytoplasmic ROS generation was measured 30 min after Khz treatment by DCF staining and flow cytometry. Left panel: Mitochondrial ROS generation was assessed 60 min after Khz treatment by MitoSOX Red staining and flow cytometry. Data represent mean ± SD.

ROS production after Khz treatment was analyzed because oxidative stress is involved in apoptosis induced by a variety of insults. Fig. 4a and b show that cytoplasmic ROS levels increased 30 min after Khz treatment. Therefore, the role of NADPH oxidase was investigated to identify the source of ROS generation after Khz treatment. Fig. 4c shows that DPI (a flavoprotein inhibitor) and apocynin (a p47^phox^ inhibitor) prevented ROS production. Moreover, silencing the expression of Nox2 and Nox4 using specific siRNAs almost completely abrogated ROS production (Fig. 4d, e). Transfection with Nox2 or Nox4 siRNA alone partially blocked ROS production, which suggests that Nox2 and Nox4 contribute to Khz-induced ROS generation together. Activation of NADPH oxidase by Khz was also demonstrated by the translocation of the cytosolic subunits of NADPH oxidase p47^phox^ and p67^phox^ to the cell membrane 15 min after Khz treatment (Fig. 4f). Taken together, these data indicate that NADPH oxidase produces ROS upon Khz treatment.

Because the mitochondrial respiratory chain is another major source of cellular ROS, mitochondrial ROS production was evaluated using MitoSOX Red staining. Mitochondrial ROS levels in HepG2 cells increased 60 min after Khz treatment (Fig. 4g); thus, it appears that ROS production by mitochondria occurs later than that by NADPH oxidase. Mitochondrial ROS generation by Khz was ceased by pretreatment with DPI or apocynin, which suggests the importance of NADPH oxidase in mitochondrial ROS production (Fig. 4h). Furthermore, the mitochondria-targeting antioxidant MitoQ did not significantly block cytoplasmic ROS generation at a concentration that completely blocked mitochondrial ROS production (Fig. 4i). TPP, which is the lipophilic moiety of MitoQ, was used as a control. Taken together, these data show that mitochondrial ROS generation induced by Khz was mediated indirectly by NADPH oxidase-derived ROS.

**Figure 5:**
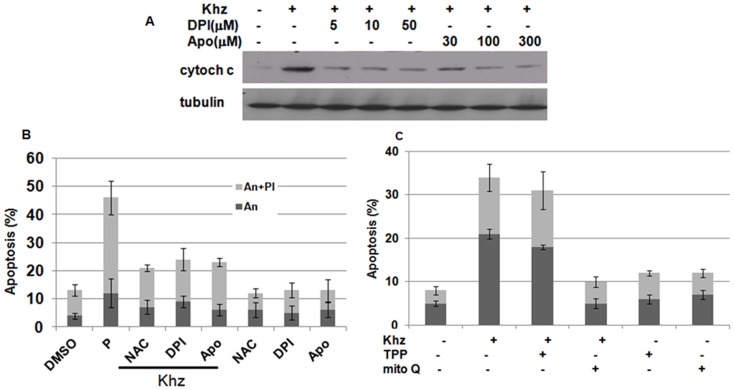
ROS generation by NADPH oxidase and mitochondria is required for Khz-induced apoptosis. (A) HepG2 cells were pretreated with DPI or apocynin for 1 h, and cytochrome c levels in the cytosol were analyzed 2 h after Khz treatment by immunoblot analysis. (B) Cells were pretreated with DPI (10 µM), apocynin (300 µM), or NAC (5 mM) for 30 min, and apoptosis was analyzed as in Fig. 2(C). (C) HepG2 cells were pretreated with 0.5 µM MitoQ or TPP for 30 min, and apoptosis was analyzed as in Fig. 2(C).

We further examined whether ROS generation through NADPH oxidase and/or mitochondria was necessary for Khz-induced apoptosis. Pretreatment of cells with DPI or apocynin suppressed mitochondrial cytochrome c release and apoptosis induced by Khz treatment, which indicates that ROS generation through NADPH oxidase was required for the induction of apoptosis (Fig. 5a, b). Furthermore, pretreatment with MitoQ, but not TPP, prevented Khz-induced apoptosis, which suggests that mitochondrial ROS generation was also necessary for the induction of apoptosis (Fig. 5c). Therefore, NADPH oxidase-derived ROS appears to trigger apoptosis *via* mitochondrial ROS generation.

### Khz induces a rapid and sustained increase in [Ca^2+^]*_i_*

**Figure 6:**
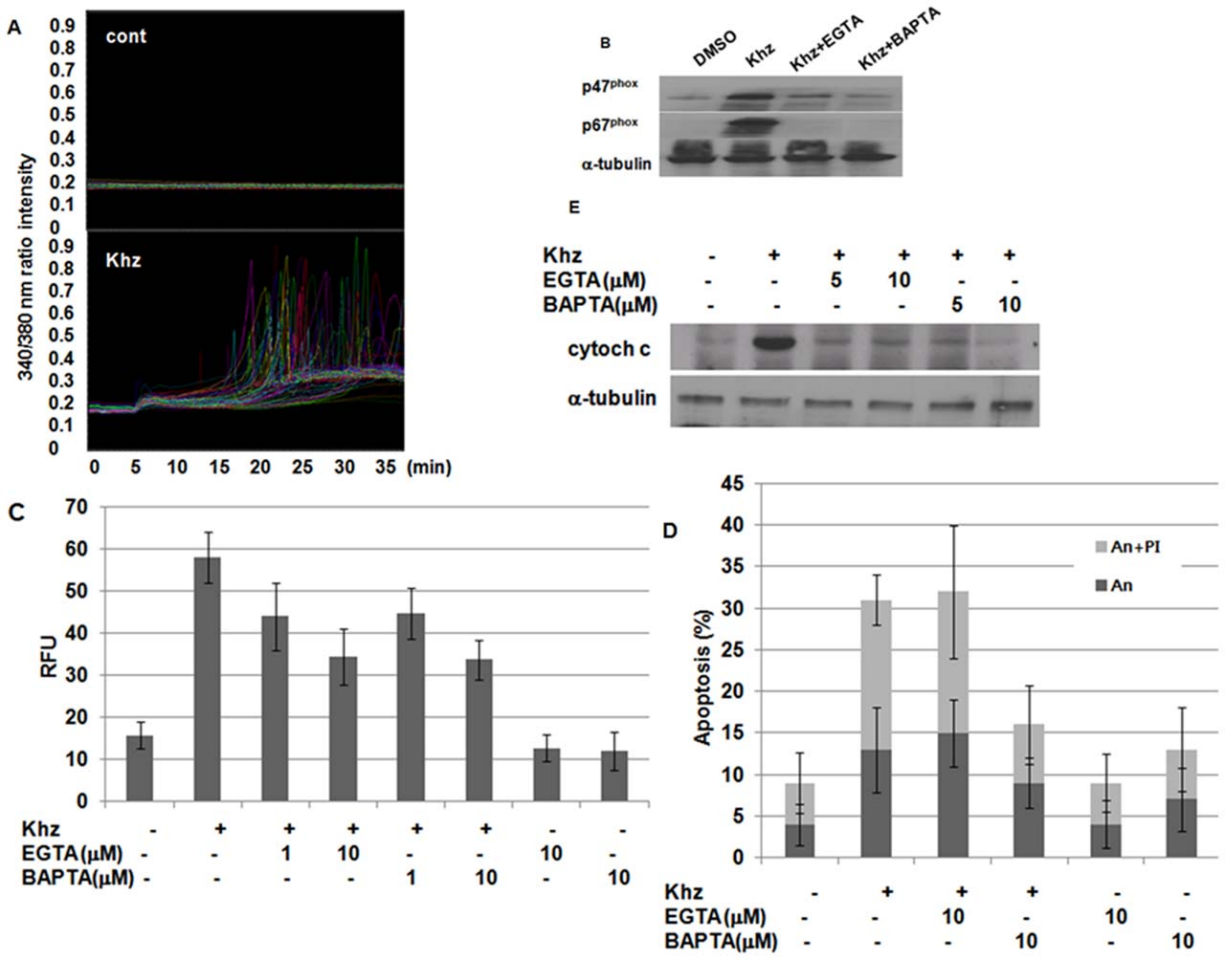
An increase of [Ca^2+^]*_i_* is necessary for Khz-induced ROS generation and apoptosis. (A) HepG2 cells were loaded with fura-2 AM for 30 min and changes in [Ca^2+^]*_i_* after Khz treatment were analyzed by digital imaging microscopy. (B) HepG2 cells were pretreated with EGTA (10 µM) or BAPTA-AM (10 µM) for 30 min, and membrane fractions were prepared for immunoblot analysis 5 min after Khz treatment. (C) Cells were pretreated with EGTA or BAPTA-AM for 30 min and treated with Khz for 30 min. ROS generation in HepG2 cells was then measured using the Amplex Red hydrogen peroxide assay. Data represent mean ± SD. (D) Cells were pretreated with EGTA or BAPTA-AM for 30 min and treated with Khz for 30 min. (E) Apoptosis was analyzed 1 h after Khz treatment as in Fig. 2(C).

Because increased [Ca^2+^]*_i_* has been widely implicated in mitochondria-mediated apoptosis, we evaluated whether Khz caused a change in [Ca^2+^]*_i_* and, if so, whether it played a role in the activation of NADPH oxidase and ROS generation. Fig. 6a shows that Khz induced an immediate and sustained increase of [Ca^2+^]*_i_* in HepG2 cells. The effects of an increase in [Ca^2+^]*_i_* on the activation of NADPH oxidase were then examined. EGTA and BAPTA-AM, which are extracellular and intracellular Ca^2+^ chelators, respectively, prevented NADPH oxidase activation as measured by the membrane translocation of the p47^phox^ and p67^phox^ subunits (Fig. 6b). Furthermore, both of these chelators inhibited ROS generation (Fig. 6c) and blocked Khz-induced mitochondrial cytochrome c release and apoptosis (Fig. 6d, e). These results indicate that Khz triggers a rapid and sustained increase in [Ca^2+^]*_i_* that, in turn, activates NADPH oxidase to induce ROS generation and, finally, apoptosis.

### JNK mediates Ca^2+^-dependent activation of NADPH oxidase

**Figure 7:**
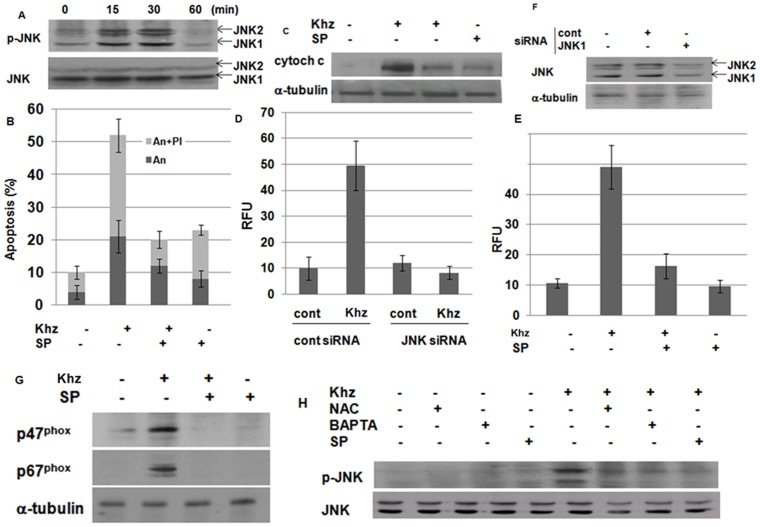
JNK mediates the increase in [Ca^2+^]*_i_* to induce NADPH oxidase-derived ROS generation. (A) Activation of JNK was analyzed in Khz-treated HepG2 cells by immunoblot analysis of phosphorylated JNK. (B) Cells were pretreated with 20 µM SP600125 (SP) for 1 h. Apoptosis was analyzed 1 h after Khz treatment as in Fig. 2(C). (C) HepG2 cells were pretreated with JNK inhibitors as in (B), and cytochrome c levels in the cytosol were analyzed by immunoblotting 2 h after Khz treatment. (D, E) HepG2 cells were transfected with control or JNK-1 siRNA. After 48 h, ROS generation was analyzed 30 min after Khz treatment using the Amplex Red hydrogen peroxide assay. Data represent mean ± SD (D). JNK protein expression was analyzed by immunoblotting 48 h after transfection (E). (F, G) HepG2 cells were pretreated with 20 µM SP200125 (SP) for 1 h, and ROS generation induced by Khz treatment was measured using the Amplex Red hydrogen peroxide assay. Data represent the mean ± SD (F). Levels of p47^phox^ and p67^phox^ protein in the membrane fractions of HepG2 cells were analyzed by immunoblotting 15 min after Khz treatment (G). (H) HepG2 cells were pretreated with NAC (5 mM), BAPTA-AM (10 µM), and SP600125 (20 µM), and JNK phosphorylation was analyzed by immunoblotting 15 min after Khz treatment.

The mitogen-activated protein kinase JNK is activated in response to oxidative stress [31]. Because Khz induced ROS generation, the activation of JNK and its possible role in Khz-induced apoptosis were examined. As shown in Fig. 7a, JNK was activated as early as 15 min after Khz treatment. Considering that ROS generation was observed 30 min after Khz treatment (Fig. 4b), it is unlikely that the activation of JNK was caused by oxidative stress. Nonetheless, JNK activity was required for the induction of apoptosis because a chemical inhibitor of JNK (SP600125) blocked mitochondrial cytochrome c release and apoptosis induced by Khz treatment (Fig. 7b, c). Inhibition of JNK by siRNA transfection or pretreatment with chemical inhibitors suppressed Khz-induced ROS production, which indicates that the activation of JNK caused ROS generation rather than the opposite (Fig. 7d–f). In agreement with these findings, pretreatment of HepG2 cells with SP600125 also suppressed the activation of NADPH oxidase, as assessed by the membrane translocation of p47^phox^ and p67^phox^ (Fig. 7g). Therefore, we examined whether JNK activation was dependent on [Ca^2+^]*_i_*. Fig. 7h shows that pretreatment with BAPTA-AM abrogated JNK activation, whereas NAC did not have a similar effect. Collectively, these results strongly indicate that JNK is activated by Khz *via* an increase in [Ca^2+^]*_i_*, thereby triggering ROS generation by NADPH oxidase and the induction of apoptosis.

## Discussion

The results of the present study show that Khz triggers caspase-dependent and mitochondria-mediated apoptotic death in human cancer cells. Khz-induced apoptosis was selective for transformed cells, and Khz showed only minimal effects on non-transformed cells, which suggests its potential as an anticancer therapeutic agent. Intrinsic oxidative stress in transformed cells may render them more susceptible to apoptosis induced by Khz. Alternatively, cancer cells may have defective restoration of glutathione (GSH) reserves. However, the GSH levels in tumor tissues are higher than those in normal tissues [32], and depleting GSH reserves often sensitizes cancer cells to ROS-induced cell death, which suggests that the GSH present in cancer cells protects them from oxidative stress.

Khz-induced apoptosis was caspase-dependent, as indicated by the activation of caspases and inhibition of apoptosis by pretreatment with the pan-caspase inhibitor z-VAD-fmk (Fig. 3a, b). The involvement of a mitochondria-mediated pathway was confirmed by the release of mitochondrial cytochrome c, and inhibition of apoptosis was confirmed by the overexpression of Bcl-2 (Fig. 3c, d). Oxidative stress is associated with apoptotic and non-apoptotic cell death, although pro-oxidative conditions are not a prerequisite for apoptosis [33]. The present study shows that the induction of apoptosis by Khz required ROS generation by both NADPH oxidase and mitochondria. Several studies have implicated ROS generated from mitochondria in the induction of apoptosis; however, the results of the present study indicate that NADPH oxidase-derived ROS were critical for Khz-induced apoptosis (Fig. 5a, b). Although mitochondrial ROS generation was also necessary for Khz-induced apoptosis (Fig. 5c), it was secondary to the initial ROS production by NADPH oxidase because the generation of mitochondrial ROS occurred after that of cytoplasmic ROS and was ceased by pretreatment with NADPH oxidase inhibitors (Fig. 4g, h). Of the 7 members of the human NADPH oxidase family, Nox2 and Nox4 were found to be responsible for ROS generation in HepG2 cells induced by Khz treatment (Fig. 4d, e).

It is widely accepted that calcium signaling plays an important role in apoptosis [27], [34]. Cross-talk between ROS and calcium signaling pathways may lead to synergistic effects on mitochondrial permeabilization and cell death. The present study showed that an increase of [Ca^2+^]*_i_* induced by Khz treatment resulted in ROS generation by NADPH oxidase. The [Ca^2+^]*_i_* increased before the generation of ROS, and Ca^2+^ chelators such as EGTA and BAPTA-AM abrogated the activation of NADPH oxidase and ROS generation (Fig. 6b, c). However, the inhibitors of NADPH oxidase had no effect on the increase of [Ca^2+^]_i_ induced by Khz treatment (data not shown). Activation of NADPH oxidase by elevated [Ca^2+^]*_i_* is well known in neutrophils and has also been reported for other cell types [35], [36].

Finally, JNK was identified as the mediator of Ca^2+^-dependent NADPH oxidase activation. JNK was activated within 15 min of Khz treatment in a Ca^2+^-dependent manner (Fig. 7a, h), and the activation occurred downstream of the increase of [Ca^2+^]*_i_* but upstream of ROS generation because it was blocked by the Ca^2+^ chelator BAPTA-AM but not by the ROS scavenger NAC (Fig. 7h). Furthermore, treatment with JNK siRNA or chemical inhibitors prevented Khz-induced ROS generation (Fig. 7d–g). Most studies place ROS upstream of JNK activation [37], [38]; however, in this study, ROS generation by NADPH oxidase was mediated by the activation of JNK.

**Figure 8:**
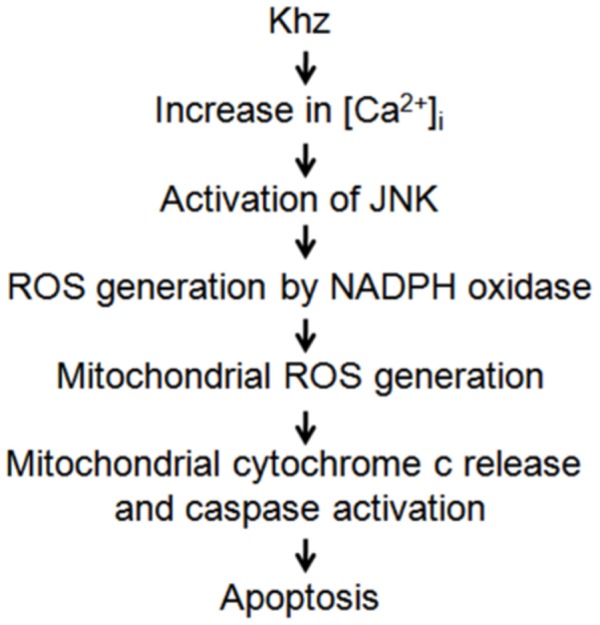
Summary of the signaling events leading to Khz-induced apoptosis.

In summary, we demonstrated in this study that Khz induces mitochondria-mediated apoptosis preferentially in transformed cells, and the signaling pathway of Khz-induced apoptosis involves an increase in [Ca^2+^]*_i_*, activation of JNK, and ROS generation through NADPH oxidase and mitochondria (Fig. 8).
